# Supplementary figures and images for: Enhanced electrophysiological recordings in acute brain slices, spheroids, and organoids using 3D high-density multielectrode arrays
Source: PLoS One. 2025 Sep 4;20(9):e0328903. doi: 10.1371/journal.pone.0328903 (PMC12410755; doi:10.1371/journal.pone.0328903)

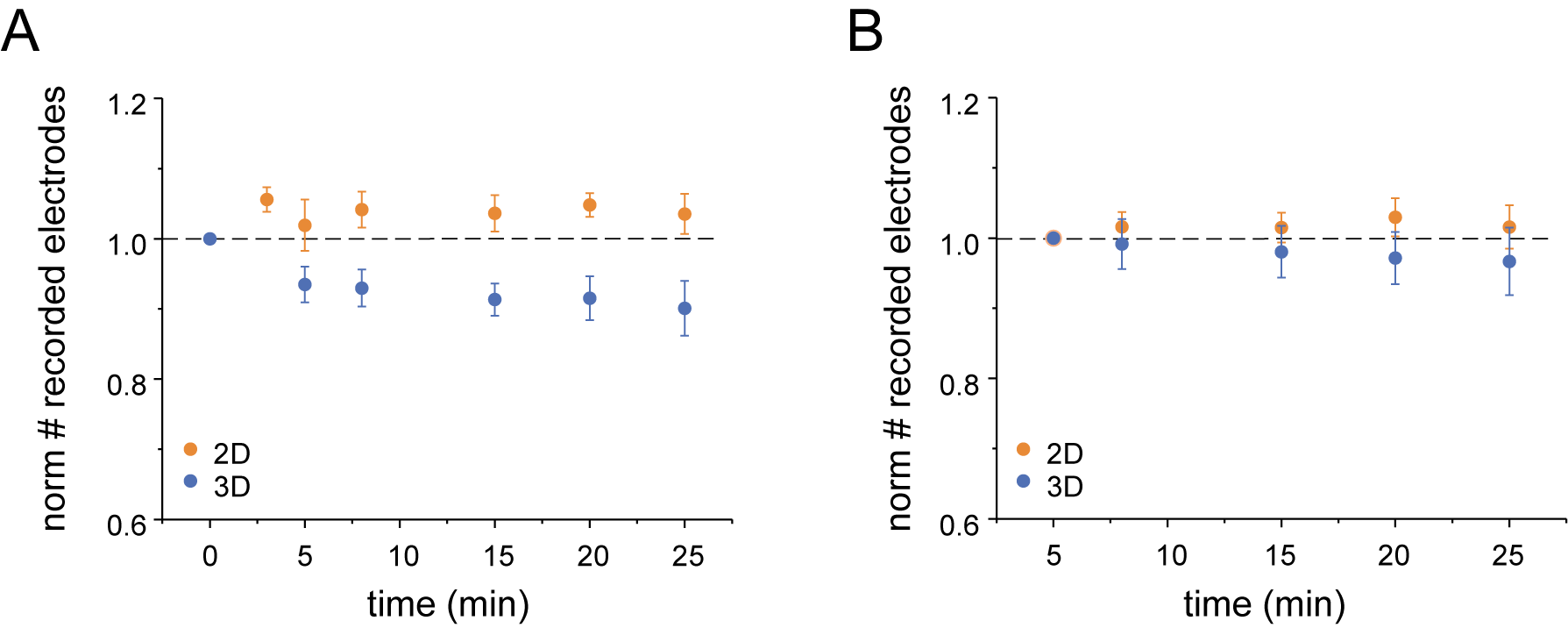

Supplement: S1 Fig — (TIF) [file pone.0328903.s001.tif]

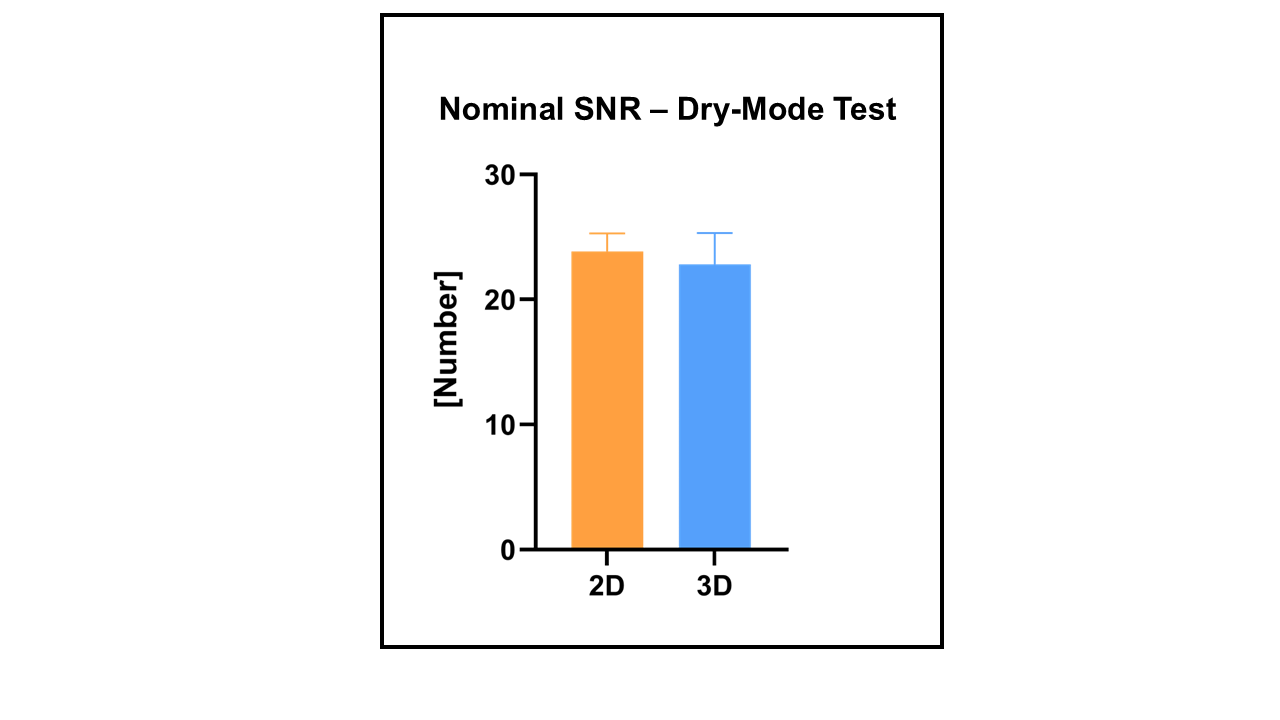

Supplement: S2 Fig — (TIF) [file pone.0328903.s002.tif]

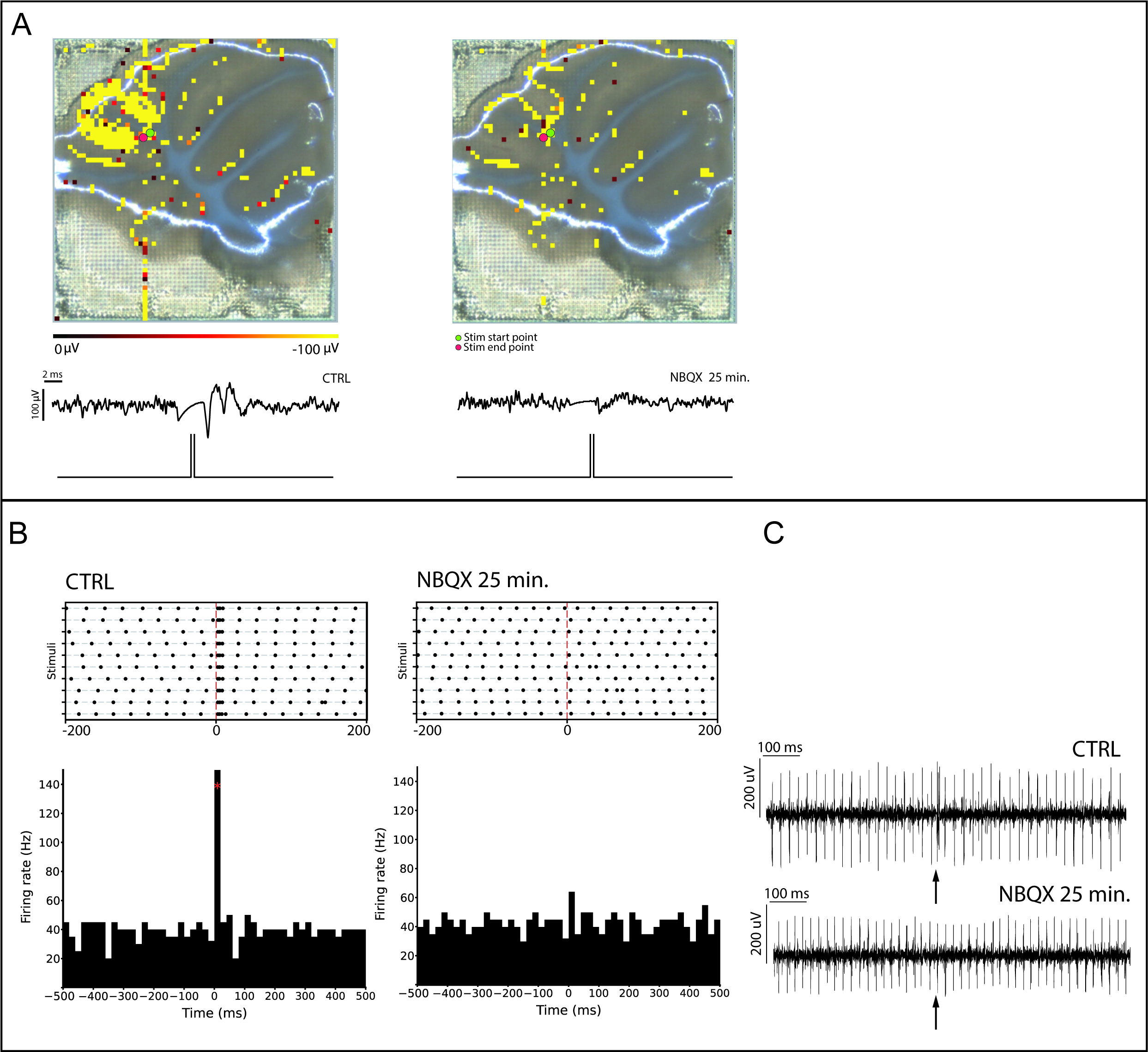

Supplement: S3 Fig — (TIF) [file pone.0328903.s003.tif]

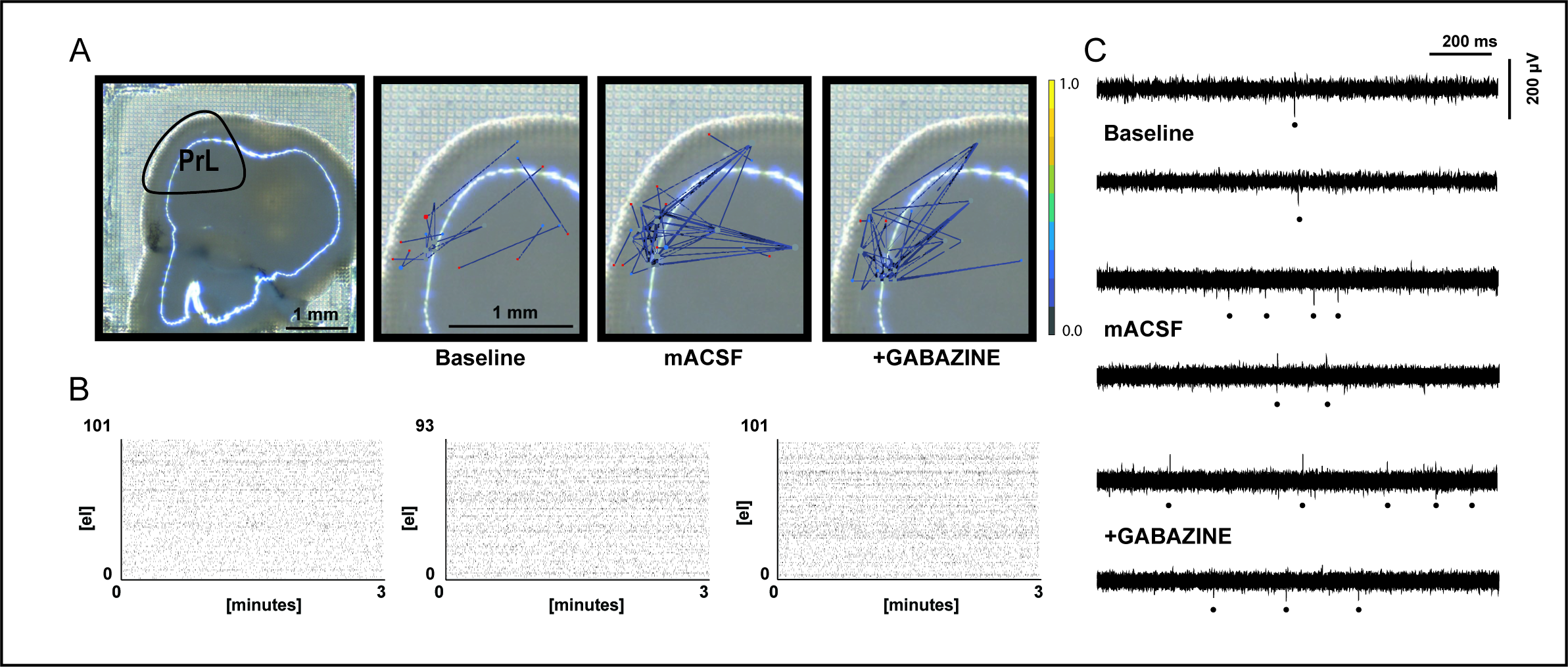

Supplement: S4 Fig — (TIF) [file pone.0328903.s004.tif]

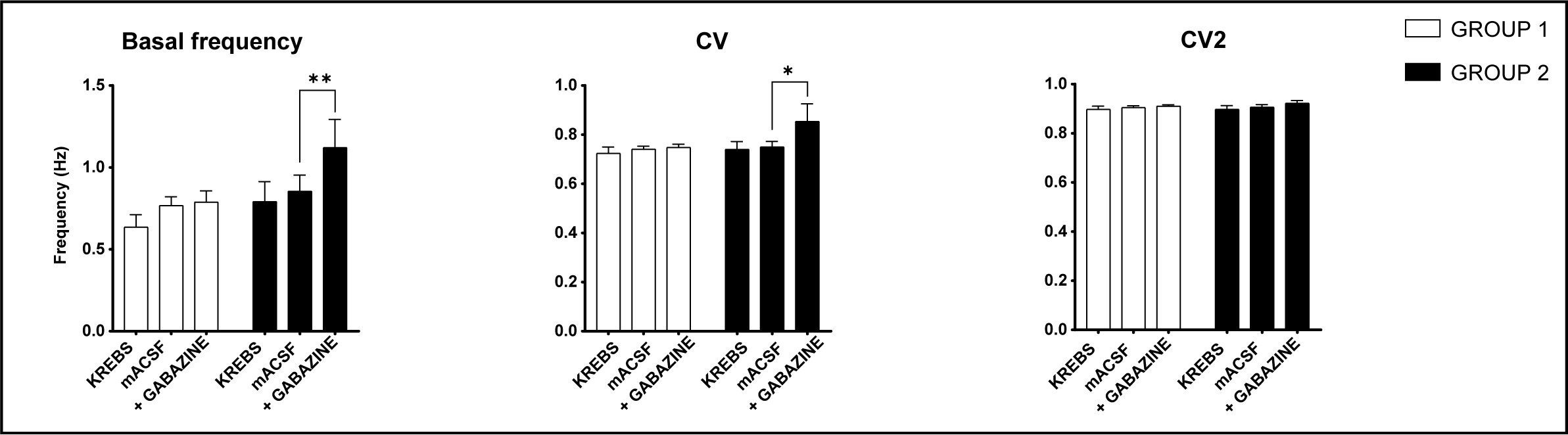

Supplement: S5 Fig — (TIF) [file pone.0328903.s005.tif]
